# Reactive-Diffusive-Advective Traveling Waves in a Family of Degenerate Nonlinear Equations

**DOI:** 10.1155/2016/5620839

**Published:** 2016-09-01

**Authors:** Faustino Sánchez-Garduño, Judith Pérez-Velázquez

**Affiliations:** ^1^Departamento de Matemáticas, Facultad de Ciencias, Universidad Nacional Autónoma de México (UNAM), Circuito Exterior, Ciudad Universitaria, 04510 Ciudad de México, Mexico; ^2^Institute of Computational Biology, Helmholtz Zentrum München, German Research Center for Environmental Health, Ingolstädter Landstraße 1, 85764 Neuherberg, Germany; ^3^Technical University of Munich, Zentrum Mathematik, M12, Boltzmannstraße 3, 85747 Garching, Germany

## Abstract

This paper deals with the analysis of existence of traveling wave solutions (TWS) for a diffusion-degenerate (at *D*(0) = 0) and advection-degenerate (at *h*′(0) = 0) reaction-diffusion-advection (RDA) equation. Diffusion is a strictly increasing function and the reaction term generalizes the kinetic part of the Fisher-KPP equation. We consider different forms of the convection term *h*(*u*): (1)  *h*′(*u*) is constant *k*, (2)  *h*′(*u*) = *ku* with *k* > 0, and (3) it is a quite general form which guarantees the degeneracy in the advective term. In Case 1, we prove that the task can be reduced to that for the corresponding equation, where *k* = 0, and then previous results reported from the authors can be extended. For the other two cases, we use both analytical and numerical tools. The analysis we carried out is based on the restatement of searching TWS for the full RDA equation into a two-dimensional dynamical problem. This consists of searching for the conditions on the parameter values for which there exist heteroclinic trajectories of the ordinary differential equations (ODE) system in the traveling wave coordinates. Throughout the paper we obtain the dynamics by using tools coming from qualitative theory of ODE.

## 1. Introduction

The strong effect produced by the addition of the nonlinear convective term *kuu*_*x*_ on the solutions behavior of the classical Fisher-KPP equation *u*_*t*_ = *u*_*xx*_ + *u*(1 − *u*) is well documented in the literature (see [1–3]). We mean those for the nonlinear reaction-diffusion-advection equation *u*_*t*_ = *u*_*xx*_ − *kuu*_*x*_ + *u*(1 − *u*). This is particularly remarkable, when the diffusion is negligible compared to the convective effects. In such a case, the solutions can exhibit shock-like behavior (see [4–6]). In the above equation the term *ku* is called the advection “speed.” It has also been proved (see [3]) that the previous equation has monotonic decreasing traveling wave solutions (TWS) *u*(*x*, *t*) = *ϕ*(*x* − *ct*) ≡ *ϕ*(*ξ*), where *c* is the speed of the wave, satisfying the boundary conditions lim_*ξ*→−*∞*_⁡*ϕ*(*ξ*) = 1, lim_*ξ*→+*∞*_⁡*ϕ*(*ξ*) = 0 with 0 < *ϕ*(*ξ*) < 1, ∀*ξ* ∈ (−*∞*, +*∞*), if and only if *c* ≥ *c*(*k*), where

(1) $$\begin{array}{c} c\left(\;k\;\right)=\left\{\begin{array}{cc} 2 & \\ \frac{k}{2}+\frac{2}{k} & \text{if }\left\{\begin{array}{cc} 2>k>-\infty & \\ 2\leq k<\infty . & \end{array}\right. \end{array}\right. \end{array}$$

In [7], the author carries out a Painlevé analysis to get some approximate solutions for the above-mentioned equation.

In a series of papers authored by Malaguti et al. [8–11], the existence of TWS of the monostable (the reaction term has two equilibria, one is asymptotically stable and the other is unstable) reaction-diffusion-convection equation,

(2) $$\begin{array}{c} u_{t}+h\left(\;u\;\right)u_{x}={\left[\;D\left(\;u\;\right)u_{x}\;\right]}_{x}+g\left(\;u\;\right), \end{array}$$

was investigated. The constant diffusion case was studied in [12] and the nondegenerate case (*D*(*u*) > 0  ∀*u* ∈ (0,1)) in [11]; they proved that (2) admits decreasing TWS.

In [8] the authors looked at the case, where *D*(*u*) is such that *D*(0) = 0, *D*(1) > 0 (simply degeneracy) and *D*(1) = 0 (double degeneracy) and *D*′(0) = 0 and *D*′(1) = 0. Although they take *h*(*u*) to be nonlinear, specific properties of *h*(*u*) are not explicitly stated. In particular, their equation is not necessarily a convection-degenerate one. Note also that even though an application of their results to the evolution of a bacterial colony is presented, this equation does not contain convection term, which makes no real application of the convection-diffusion problem. In [10] continuous dependence of the threshold wave speed and of the traveling wave profiles is studied with respect to the diffusion, reaction, and convection terms. In [12] degenerate convection was considered. More recently the authors considered aggregation (i.e., the diffusion term changes sign) [9], *D*(*u*) > 0 for *u* ∈ (0, *α*), *D*(*u*) < 0, for *u* ∈ (*α*, 1), and a bistable term [13]. See [14] for a review.

Gilding and Kersner [15] and separately Mansour [16] looked at the particular case *u*_*t*_ + *bu* ^*k*^ *u*_*x*_ = (*au* ^*k*^ *u*_*x*_)_*x*_ + *cu*(1 − *u* ^*k*^) arising in the study of pattern formation by bacterial colonies. Here *a* ≥ 0, *b*, *c* ≥ 0, and *k* > 0 are constants. Kamin and Rosenau [17] also looked at a similar special case, with *D*(*u*) = *h*(*u*) = *u* ^*m*^ and *g*(*u*) = *u*(1 − *u*)^*m*−1^. In both papers, the set of wave speeds from which the equation admits a wavefront are studied.

The incorporation of a more general nonlinear advection term in the above equation also has been discussed in [3]. In such case, that equation takes the form *u*_*t*_ = *u*_*xx*_ − [*h*(*u*)]_*x*_ + *u*(1 − *u*), where the convection “speed” is *h*′(*u*).

The aim of this paper is the investigation of the existence of TWS for the one-dimensional nonlinear degenerate RDA equation

(3) ∂u∂t=∂∂xDu∂u∂x−∂∂xhu+gu∀x,t∈R×R+,

where the functions *D*, *h*, and *g* are defined on the interval [0,1] and there, they satisfy the following conditions:

(1) *D* ∈ *C*_[0,1]_^2^ with *D*(0) = 0, *D*(*u*) > 0  ∀*u* ∈ (0,1]; *D*′(*u*) > 0  ∀*u* ∈ [0,1] and *D*′′(*u*) ≠ 0  ∀*u* ∈ [0,1].
(2) *h* ∈ *C*_[0,1]_^1^ with *h*′(*u*) > 0  ∀*u* ∈ (0,1]. Two cases will be considered for *h*′(0). Namely,
  (a) *h*′(0) > 0,
  (b) *h*′(0) = 0.
(3) *g* ∈ *C*_[0,1]_^2^ with *g*(0) = *g*(1) = 0, *g*(*u*) > 0  ∀*u* ∈ (0,1); *g*′(0) > 0 and *g*′(1) < 0.

The degeneracy (at *u* = 0) could have two sources: the diffusion term *D*(0) = 0 and the case of item (2)(b), for the advective term, where *h*′(0) = 0.

The inclusion of the first-order spatial derivative term *h*′(*u*)*u*_*x*_ in (3) transforms the parabolic degenerate nature of (3) with *h*′(*u*) ≡ 0 into a hyperbolic-like type. In fact, given that the partial derivative ∂*F*/∂*u*_*xx*_ = *D*(*u*) of the nonlinear operator

(4) Fu,ux,uxx≡Duuxx+D′uux2−h′uux+gu,

vanishes at *u* = 0, *F* is not elliptic precisely at *u* = 0. Because of that, the nonlinear operator,

(5) $$\begin{array}{c} L\left[\;u\;\right]\equiv F\left(\;u,u_{x},u_{xx}\;\right)-u_{t}, \end{array}$$

is not parabolic at *u* = 0. See [18].

The degeneracy of the equation involves two important features of its solutions. One is the* finite speed of propagation* throughout the space. The other is that, for general rule, we do not expect that all the initial and boundary conditions problem associated with (3) possesses a classical solution, that is, smooth enough solution.

The TWS analysis we carried out through this paper uses a dynamical systems approach, which is different to that used by other authors [12, 15] and focuses on the qualitative behavior of the trajectories of a phase portrait as the involved parameters change. Additionally, in order to show the TWS whose existence we prove, we numerically solve the initial and boundary value problems associated with the full RDA in each considered case.

In ecological terms, (3) could describe the space-temporal dynamics of one species living in a one-dimensional habitat subject to the following factors: a density-dependent diffusion term *D*(*u*) which produces a pressure on the individuals of the population to migrate from crowded areas to sparse ones (for more details on this interpretation of *D*(*u*), see [19] and references therein), a nonlinear advective term *h*′(*u*) which “pushes” the population towards the direction −*u*_*x*_ (a sort of “directed wind”; see [3]), and a density-dependent growth rate *g*(*u*) which, by its qualitative features given in item (3), gives the dynamics of a habitat with limited resources (logistic growth). The carrying capacity of the habitat, in nondimensionalized form, is one.

The derivation of (3) can be done by using the microscopic individual behavior (random walks approach) which can be seen somewhere else (see [20] or [21]). Here we are omitting the details.

Note that taking *h*′(*u*) = *V*(*u*) + *uV*′(*u*), where *A* is an arbitrary constant, we obtain the equation mentioned and studied in [22].

Among the possible space-time patterns which could be described by (3), are those of traveling wave type, that is, solutions of the form *u*(*x*, *t*) = *ϕ*(*x* − *ct*), where *c* is the wave speed. These can be interpreted as waves of invasion of the population into the habitat.

Our analysis is based on the assumption that to look for TWS in a functional space is equivalent to search the set of parameters (in which the speed *c* is included) for which a two-dimensional system of ODE possesses heteroclinic trajectories. This system comes from the restatement of the original problem into the appropriate traveling wave variable.

The TWS analysis for (3) we present in this paper will be carried out in stages corresponding to the different levels of complexity the function *h*′ exhibits. These are the cases we consider.

**Case 1 .**

*h*′(*u*) = *k*.

**Case 2 .**

*h*′(*u*) = *ku*.

**Case 3 .**

No specific form for *h*′(*u*). This function must satisfy the quite basic requirements as stated in item (2).

These three cases are studied separately. Thus, the analysis of each one is the contents of the following three sections of this paper.

## 2. The TWS Analysis for *h*′(*u*) = *k*

With the choice *h*′(*u*) = *k*, the RDA equation (3) becomes

(6) $$\begin{array}{c} \frac{\partial u}{\partial t}=\frac{\partial }{\partial x}\left[\;D\left(\;u\;\right)\frac{\partial u}{\partial x}\;\right]-k\frac{\partial u}{\partial x}+g\left(\;u\;\right), \end{array}$$

where the density-dependent diffusion coefficient *D* and the kinetic part *g* satisfy the conditions listed in the previous section.

Note that because of the qualitative features of *D* and *g* on the interval [0,1], the pair of functions *u*_0_(*x*, *t*) ≡ 0 and *u*_1_(*x*, *t*) ≡ 1 are homogeneous and stationary solutions of (6) for all (*x*, *t*) ∈ *ℝ* × *ℝ* ^+^. Their role in choosing the boundary condition for the TWS of (6) will be clear later.

### 2.1. A Quick Review on the *k* = 0 Case

For *k* = 0, (6) becomes

(7) $$\begin{array}{c} \frac{\partial u}{\partial t}=\frac{\partial }{\partial x}\left[\;D\left(\;u\;\right)\frac{\partial u}{\partial x}\;\right]+g\left(\;u\;\right). \end{array}$$

The traveling wave dynamics of (7) has been already studied (see [19]). In this reference, the authors proved that for *D* and *g* satisfying the conditions stated before, there exists a unique value, *c* ^*∗*^ > 0, of *c*, such that (7) has

(1) no traveling wave solutions for 0 < *c* < *c* ^*∗*^,
(2) a unique traveling wave solution of sharp type for *c* = *c* ^*∗*^,
(3) a traveling wave solution of smooth decreasing front type satisfying the boundary conditions lim_*ξ*→−*∞*_⁡*ϕ*(*ξ*) = 1 and lim_*ξ*→+*∞*_⁡*ϕ*(*ξ*) = 0 with 0 < *ϕ*(*ξ*) < 1  ∀*ξ* ∈ (−*∞*, +*∞*), for each *c* > *c* ^*∗*^.

The profile of the TWS mentioned in items (2) and (3) can be found in [19].

### 2.2. The Analysis for *k* ≠ 0

In this subsection, we investigate the existence of decreasing TWS for (6). The specific TWS satisfy the boundary conditions lim_*ξ*→−*∞*_⁡*ϕ*(*ξ*) = 1 and lim_*ξ*→+*∞*_⁡*ϕ*(*ξ*) = 0 with 0 < *ϕ*(*ξ*) < 1  ∀*ξ* ∈ (−*∞*, +*∞*).

Our analysis starts with the physical interpretation of the convective term −*ku*_*x*_ in (6). The diffusive substance is pushed out with speed *k* towards the direction of −*u*_*x*_.

Suppose that $u(x,t)=\phi (x-(\tilde{c}+k)t)$ is a traveling wave solution of (6) satisfying the appropriate boundary conditions. We then set $\xi_{1}=x-(\tilde{c}+k)t$, by substituting $u(x,t)=\phi (x-(\tilde{c}+k)t)\equiv \phi (\xi_{1})$ into (6); we obtain the second-order ODE:

(8) $$\begin{array}{c} -\left(\;\tilde{c}+k\;\right)\phi^{\prime }=D\left(\;\phi \;\right)\phi^{\prime \prime }+D^{\prime }\left(\;\phi \;\right){\left[\;\phi^{\prime }\;\right]}^{2}+g\left(\;\phi \;\right), \end{array}$$

where the symbol  ′ on *ϕ* means derivative respect *ξ*_1_ and on *D* means derivative with respect to *ϕ*. Note that, denoting by $c=\tilde{c}+k$, actually (8) is of the form

(9) $$\begin{array}{c} -c\phi^{\prime }=D\left(\;\phi \;\right)\phi^{\prime \prime }+D^{\prime }\left(\;\phi \;\right){\left[\;\phi^{\prime }\;\right]}^{2}+g\left(\;\phi \;\right). \end{array}$$

This second-order ODE, except that the derivative  ′ is with respect to *ξ*_1_, has exactly the same form as the corresponding second-order ODE in the traveling wave coordinate for the TWS for (7) (see [19]). The following argument justifies this simplification. Let *T* : *ℝ* ^2^ → *ℝ* ^2^ be a linear transformation such that for (*x*, *t*) ∈ *ℝ* × *ℝ* ^+^

(10) $$\begin{array}{c} T\left(\;x,t\;\right)=\left(\;x-kt,t\;\right)\equiv \left(\;x^{\prime },t\;\right). \end{array}$$

The following proposition holds. Let *w*(*x*′, *t*) be such that *u*(*x*, *t*) = *w*(*x*′, *t*).

**Proposition 1 .**

If *u*(*x*, *t*) is solution of (6), then *w*(*x*′, *t*) satisfies

(11) $$\begin{array}{c} \frac{\partial w}{\partial t}=\frac{\partial }{\partial x^{\prime }}\left[\;D\left(\;w\;\right)\frac{\partial w}{\partial x^{\prime }}\;\right]+g\left(\;w\;\right). \end{array}$$

**Proof**

This follows by using the chain rule; we obtain *u*_*t*_ = −*kw*_*x*′_ + *w*_*t*_ and *u*_*x*_ = *w*_*x*′_. Then we substitute *u*_*t*_ and *u*_*x*_ into (6) to arrive to the above equation.

**Remark 2 .**

Formally, the meaning of Proposition 1 is as follows: the convective effect in (6) can be suppressed by simply traveling in a space moving system of coordinates, which moves parallel to the *x*-axis with the convective speed *k*.

Hence, in the light of the previous reasoning the existence of TWS analysis for (6) is essentially reduced to the methodology developed in [19]. Therefore, by adapting and reinterpretation of the results given there, the following proposition holds in the present case.

**Proposition 3 .**

If the functions *D* and *g* satisfy the conditions (1) and (3) and *ϕ*(*x*, *t*) is solution of (7) on −*∞* < *x* < +*∞*, *t* > 0, then for each *k* ≠ 0, *ψ*(*x*, *t*) ≡ *ϕ*(*x* − *kt*, *t*) is solution of (6).

**Proof**

This follows by using the hypothesis and the chain rule. In fact, by hypothesis for each (*x*, *t*) such that −*∞* < *x* < +*∞*, *t* > 0

(12) $$\begin{array}{c} \frac{\partial \phi }{\partial t}={\left[\;D\left(\;\phi \;\right)\frac{\partial \phi }{\partial x}\;\right]}_{x}+g\left(\;\phi \;\right). \end{array}$$

Computing the relevant derivatives

(13) ψt=−cϕξ+ϕt,ψξ,ψxx=ϕξξ,

and using (8) and (9) appropriately, we obtain the equation for which *ψ* is solution, as stated in the proposition.

**Figure 1:**
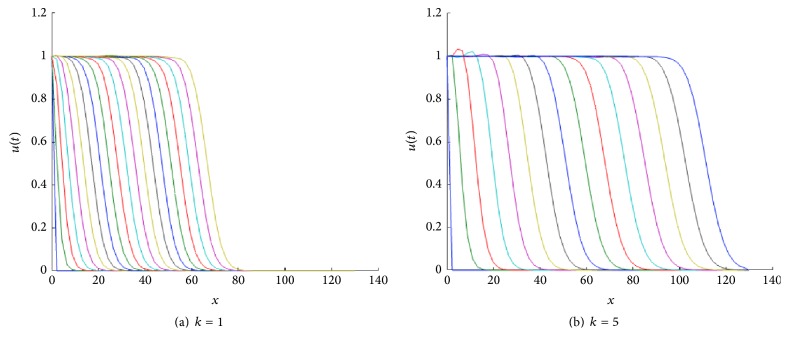
Front traveling waves solutions of (6) at equal time intervals *t* = 1,2, 3,… with *g*(*u*) = *u*(1 − *u*) and *D*(*u*) = *u*(1.0 + *βu*) for *β* = 1. The values for *k* are indicated in each figure. The initial condition used was *u*(*x*, 0) = 0.5(1.0 + tanh⁡((0.4 − *x*)/0.1)), over the spatial domain is 0 < *x* < 140.

In Figure 1 we show the corresponding traveling wave profiles for two values of *k*. These were obtained by numerically solving (6) with the numerical routine NAG P03PCF (NAG Matlab Toolbox). As initial conditions we took 0.5(1.0 + tanh⁡((0.4 − *x*)/0.1)).

**Figure 2:**
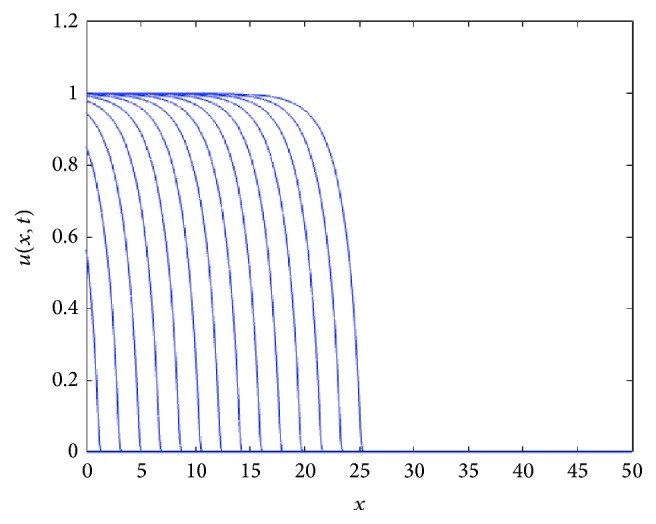
Evolution of a compact support function as initial condition under the reaction-diffusion convection process described by (6). The numerics show that as the time grows, the approximative solution tends to the sharp traveling wave solution. Note the agreement between the calculated speed of the sharp solution with that for which we have the saddle-saddle heteroclinic trajectory; see Figure 3. The profiles are plotted at equal time intervals *t* = 1,2, 3,… with *g*(*u*) = *u*(1 − *u*) and *D*(*u*) = *u*(1.0 + *βu*) for *β* = *k* = 1, compact support initial conditions (in a larger domain −50 < *x* < 40, *u*(*x*, 0) = 1 for *x* < −20, and *u*(*x*, 0) = 0 for *x* ≥ 20).

For *k* > 0, the speed of the TWS of (6) is faster than the TWS when *k* = 0. For negative *k*, the speed is slower than TWS when *k* = 0. In particular, this is true for the sharp type solution. See in Figure 2 an illustration of how the sharp type traveling wave evolves. The sharp traveling wave shown here (and in all remaining cases through the paper) was computed using the PDE numerical solver of Matlab (pdepe) using compact support initial conditions (in a larger domain −50 < *x* < 40, *u*(*x*, 0) = 1 for *x* < −20 and *u*(*x*, 0) = 0 for *x* ≥ 20). Note that the issue of accurately numerically computing a degenerate traveling wave represents on its own a research topic in numerical analysis; this is beyond the scope of this paper and we refer the interested reader to [23].

**Figure 3:**
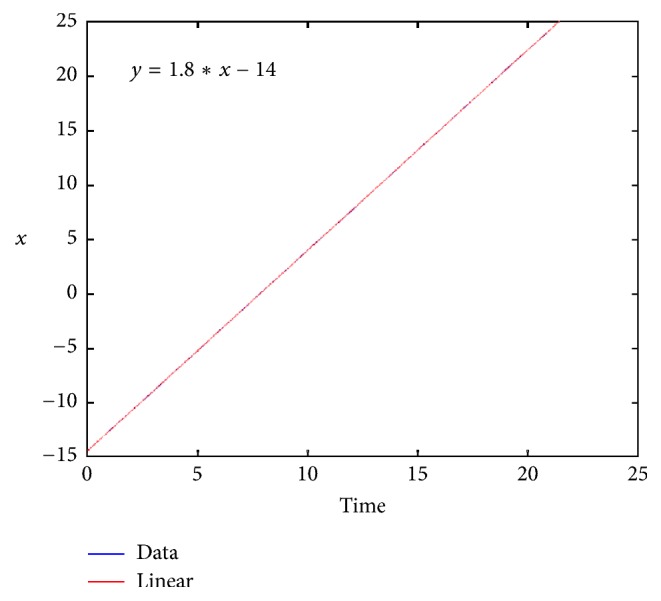
Approximation of the wave speed of the profiles evolving to a sharp traveling wave; to compute this we follow how the wave's location changes with each time step. For a fixed value *u* ≡ 0.5 we compute *x*_1_, *x*_2_,… so that *x*_*i*_ is the *x*, where *u*(*x*, *t*_*i*_) = 0.5. We plot this curve versus time and compute the slope to approximate the wave speed and obtain 1.8 as a result. For these parameter values (*β* = *k* = 1), the phase portrait numerical results show that the value of *c* for which we have a unique heteroclinic connexion between *P*_1_ and *P*_*c*_ is for *c* ^*∗*^ ≈ 1.82.

We also computed numerically the speed of the sharp wave (see Figure 3). To compute this we follow how the wave's location changes with each time step; that is for a fixed value *u* ≡ 0.5 we compute *x*_1_, *x*_2_,… so that *x*_*i*_ is *x*, where *u*(*x*, *t*_*i*_) = 0.5. We plot this curve versus time and compute the slope to approximate the wave speed; we have obtained 1.8 as a result. We note that the corresponding sharp wave for *k* = 0 has a speed of *c*_*k*=0_^*∗*^ ≈ 0.8; that is, when *k* = 1, *c*_*k*=1_^*∗*^ = *c*_*k*=0_^*∗*^ + 1 = 1.8, making an illustrative example of Proposition 3. Furthermore, for these parameter values (*β* = *k* = 1), the phase portrait numerical results show that the value of *c* for which we have a unique heteroclinic connexion between *P*_1_ and *P*_*c*_ (corresponding to the sharp traveling wave) is *c* ^*∗*^ ≈ 1.82.

## 3. Traveling Wave Solutions Analysis with *h*′(*u*) = *ku*

Here, (3) takes the form

(14) ∂u∂t=∂∂xDu∂u∂x−ku∂u∂x+gu,∀x,t∈R×R+,

where the functions *D* and *g* satisfy conditions (1) and (3) of Section 1. As in the equation analyzed in Section 2, the functions *u*_0_(*x*, *t*) ≡ 0 and *u*_1_(*x*, *t*) ≡ 1 are homogeneous and stationary solutions of (14) for all (*x*, *t*) ∈ *ℝ* × *ℝ* ^+^.

We will impose the same conditions as in Section 2 for the possible TWS of (14). The TWS analysis we are going to do starts by assuming the existence of positive *c* and *ϕ* such that *u*(*x*, *t*) = *ϕ*(*x* − *ct*) ≡ *ϕ*(*ξ*) is solution of (14). Hence, by substituting *u*(*x*, *t*) = *ϕ*(*x* − *ct*) into (14), we get the nonlinear second-order ODE:

(15) −cϕ′ξ=Dϕϕ′′ξ+D′ϕϕ′ξ2−kϕξϕ′ξ+gϕ,

which, by setting *v* = *ϕ*′, can be written as the* singular* (at *ϕ* = 0) two-dimensional ODE system:

(16) ϕ′=v,Dϕv′=kϕ−cv−D′ϕv2−gϕ.

The singularity can be removed by using a standard reparametrization of (16) (see [19]). Thus, by introducing the new parameter *τ* such that for *ϕ* > 0,

(17) $$\begin{array}{c} \frac{d\tau }{d\xi }=\frac{1}{D\left(\phi \left(\xi \right)\right)}, \end{array}$$

we obtain the nonsingular system:

(18) ϕ˙=Dϕv≡f1ϕ,v,v˙=−c−kϕv−D′ϕv2−gϕ≡f2ϕ,v,

where the dot on *ϕ* and *v* denotes the derivative of these variables with respect to *τ*. This new system is topologically equivalent to (16) in the region

(19) $$\begin{array}{c} F=\left\{\;\left(\;\phi ,v\;\right)\;|\;0<\phi \leq 1,\;-\infty <v<+\infty \;\right\}. \end{array}$$

In this region, for positive *c* system (18) has three equilibria: *P*_0_ = (0,0), *P*_1_ = (1,0), and *P*_*c*_ = (0, −*c*/*D*′(0)). Hence, according to the conditions of interest, the problem of showing the existence of the TWS satisfying such conditions in the full nonlinear PDE transforms into a dynamical systems problem. This is searching for the existence of the parameter values for which there exist heteroclinic trajectories of (18) connecting *P*_1_ with *P*_0_ or with *P*_*c*_. The analysis is conducted by stages.

### 3.1. Local Dynamics

First, let us determine the local behavior of the trajectories of (18) in a neighborhood of each equilibria. For this aim, we obtain the Jacobian matrix of the vector field defined in (18) at any point (*ϕ*, *v*). This is

(20) Jf1,f2ϕ,v=D′ϕvDϕkv−D′′ϕv2−g′ϕ−c−kϕ−2D′ϕv.

The evaluation of (20) at *P*_0_ gives us

(21) $$\begin{array}{c} J{\left[\;f_{1},f_{2}\;\right]}_{\left(0,0\right)}=\left[\begin{array}{cc} 0 & 0\\ -g^{\prime }\left(0\right) & -c \end{array}\right], \end{array}$$

from which we have tr⁡*J*[*f*_1_, *f*_2_]_(0,0)_ = −*c* < 0 for all positive values of *c* and det⁡*J*[*f*_1_, *f*_2_]_(0,0)_ = 0. Given that the eigenvalues of (21) are *λ*_1_ = 0 and *λ*_2_ = −*c*, then *P*_0_ is a* nonhyperbolic point* of* codimension one* (see [24]). The corresponding eigenvectors are **v**_1_ = (−*c*/*g*′(0), 1) and **v**_2_ = (0,1).

Because the Hartman-Grobman Theorem is not applicable here, the local dynamics of (18) around *P*_0_ does not follow from the corresponding linear approximation. In such a case, we must use the higher order terms in the Taylor series of the vector field (*f*_1_, *f*_2_) around *P*_0_. In fact, we should obtain the normal form of (18) and then use the Center Manifold Theorem (see [24]). This tells us the local dynamics of (18) around *P*_0_ can be essentially reduced to that around its center manifold. By proceeding as we already mentioned it, we conclude that *P*_0_ is a* saddle-node* point (see [19] for a similar analysis).

Evaluating (20) at *P*_1_ we obtain the Jacobian matrix

(22) $$\begin{array}{c} J{\left[\;f_{1},f_{2}\;\right]}_{\left(1,0\right)}=\left[\begin{array}{cc} 0 & D\left(1\right)\\ -g^{\prime }\left(1\right) & -\left(c-k\right) \end{array}\right]. \end{array}$$

From here, tr⁡*J*[*f*_1_, *f*_2_]_(1,0)_ = −(*c* − *k*) and det⁡*J*[*f*_1_, *f*_2_]_(1,0)_ = *g*′(1)*D*(1) < 0; therefore *P*_1_ is a hyperbolic saddle point for all positive values of *c* and *k*. The corresponding eigenvalues and the eigenvectors are

(23) λ1=−c−k+c−k2−4D1g′12,λ2=−c−k−c−k2−4D1g′12,v1=−c−k+c2−2kc+k2−4D1g′12g′1,1,v2=−c−k−c2−2kc+k2−4D1g′12g′1,1,

respectively.

At *P*_*c*_, (20) reduces to

(24) Jf1,f20,−c/D′0=−c0−kcD′0−D′′0D′20c2−g′0c.

Then, it follows tr⁡*J*[*f*_1_, *f*_2_]_(0,−*c*/*D*′(0))_ = 0 and det⁡*J*[*f*_1_, *f*_2_]_(0,−*c*/*D*′(0))_ = −*c* ^2^ < 0; therefore *P*_*c*_ is a hyperbolic saddle point for all *c* ≠ 0 and *k*. The eigenvalues and eigenvectors are *λ*_1_ = −*c*, *λ*_2_ = *c*; **v**_1_ = (2*cD*′(0)^2^/(*D*′′(0)*c* ^2^ + *kD*′(0)*c* + *D*′(0)^2^ *g*′(0)), 1) and **v**_2_ = (0,1), respectively.

### 3.2. The Nullclines: Towards the Global Dynamics

In order to study the global dynamics associated with system (18), we should understand how its nullclines behave as the involved parameters change. The horizontal nullclines of system (18) are the horizontal and the vertical axis of the *ϕv* plane. The vertical nullcline has these two branches

(25) V1ϕ=−c−kϕ+c−kϕ2−4D′ϕgϕ2D′ϕ,V2ϕ=−c−kϕ−c−kϕ2−4D′ϕgϕ2D′ϕ.

From its respective expression, it follows

(26) V10=V11=0,V20=−cD′0,V21=−c−kD′1.

Note that for all positive *c*, *V*_2_(0) < 0; meanwhile, given the positiveness of *D*′(1), the sign of *V*_2_(1) changes according with the sign of the term −(*c* − *k*).

### 3.3. Dynamics for Extreme Values of *c*

Here we are going to analyze the dynamics of (18) by considering extreme (including *c* = 0) values of *c*. This is done by considering two separate cases.

#### 3.3.1. For *c* = 0

For *c* = 0 system (18) becomes

(27) ϕ˙=Dϕv,v˙=kϕv−D′ϕv2−gϕ,

whose equilibrium points (in the region of interest) are *P*_0_ = (0,0) and *P*_1_ = (1,0). Here *P*_0_ comes from the collapse of *P*_*c*_ into the origin and this point becomes a nonhyperbolic equilibrium of* codimension two*; meanwhile *P*_1_ stills as a hyperbolic saddle point.

The vertical nullcline branches of system (27) are

(28) V1ϕ=kϕ+kϕ2−4D′ϕgϕ2D′ϕ,V2ϕ=kϕ−kϕ2−4D′ϕgϕ2D′ϕ.

**Figure 4:**
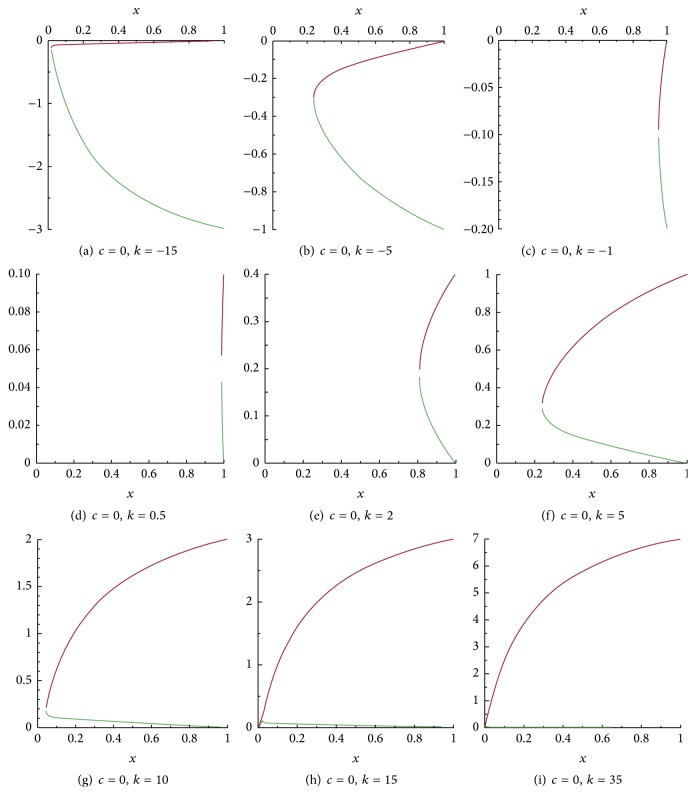
The branches of the vertical nullcline of system (27) for *c* = 0 and several values of *k*. Here *β* = 2.

Figure 4 contains a panel of figures illustrating the behavior of (28) in a representative case, where *D*(*u*) = *u* + *βu* ^2^ and *g*(*u*) = *u*(1 − *u*) for fixed positive *β* and different values of *k*.

As it can be seen in Figure 4, for small values of *k*, *V*_1_ and *V*_2_ (given by (28)) are not defined on the whole interval [0,1]. They are, however, well defined on this interval for big enough values of *k* and fixed positive *β*. Moreover, the behavior of (28)—according to Figure 4—can be classified in three main categories. These are illustrated in each row of the mentioned figures.

For each positive *k*, let us introduce the following notation:

(29) $$\begin{array}{c} v_{k}\left(\;\phi \;\right)=\frac{k\phi }{D^{\prime }\left(\phi \right)}. \end{array}$$

The following proposition holds.

**Proposition 4 .**

For each positive *k* system (27) does not have closed trajectories on the following sets:

(1) {(*ϕ*, *v*)∣0 ≤ *ϕ* ≤ 1, −*∞* < *v* < 0},
(2) {(*ϕ*, *v*)∣0 ≤ *ϕ* ≤ 1, *v* < *v*_*k*_(*ϕ*)},
(3) {(*ϕ*, *v*)∣0 ≤ *ϕ* ≤ 1, *v* > *v*_*k*_(*ϕ*)}.

**Proof**

This follows by a straightforward application of Bendixson's Negative Criterion. In fact, the divergence, div, of the vector field which defines system (27) is

(30) $$\begin{array}{c} \mathrm{div}\vec{F}\left(\;\phi ,v\;\right)=k\phi -D^{\prime }\left(\;\phi \;\right)v. \end{array}$$

Given *D* is a strictly increasing function on [0,1], for item (1)  $\mathrm{div}\vec{F}(\phi ,v)$ is positive, the same sign for item (2); meanwhile $\mathrm{div}\vec{F}(\phi ,v)<0$ in the third case. Then the proof follows.

As a consequence of Proposition 4 and the Poincaré-Bendixson Theorem on each set this proposition states the *ω* and *α* limit sets of the trajectories are equilibrium points.

In what follows we are going to use the behavior of both branches of the vertical nullcline for the determination of the phase portrait of system (27).

**Proposition 5 .**

For small enough positive values—including *c* = 0—of *c* system (18) does not have nontrivial heteroclinic trajectory in the strip {(*ϕ*, *v*)∣0 ≤ *ϕ* ≤ 1, −*∞* < *v* < +*∞*}.

**Proof**

By trivial heteroclinic trajectories, we mean those of (18) for which *ϕ* = 0  ∀*τ* ∈ (−*∞*, *∞*), as it is the case for each trajectory running on the negative vertical axis of the phase portrait, connecting *P*_*c*_ with *P*_0_ which exists for all *c* > 0. Because of the physical interpretation of *ϕ*, we are not interested in those.

**Figure 5:**
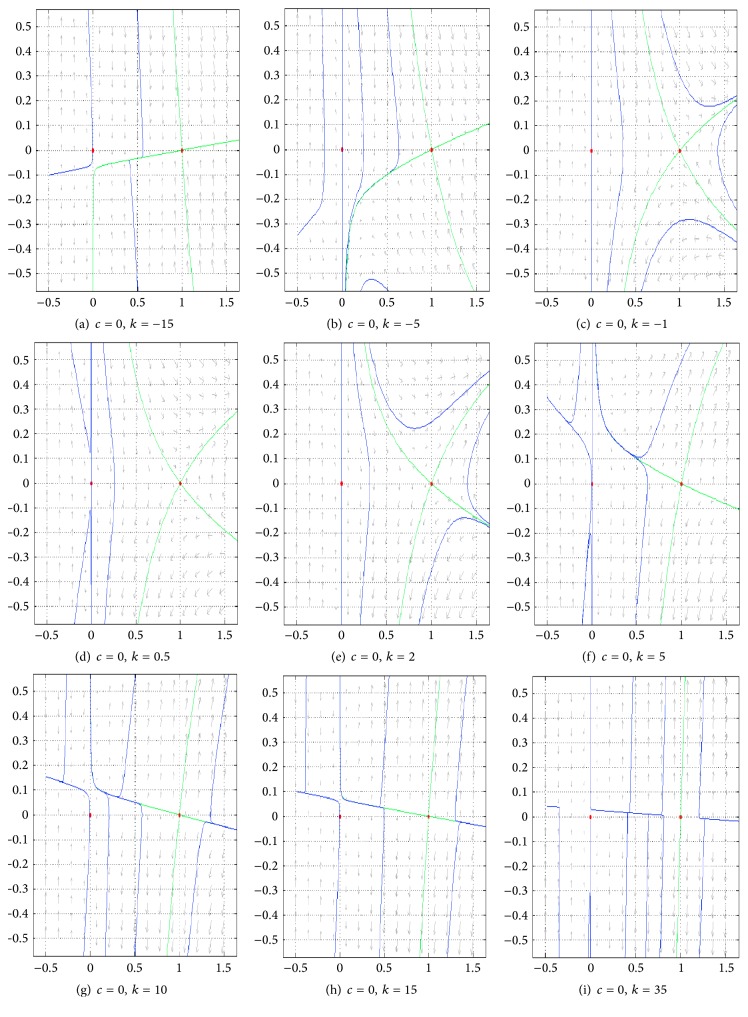
Phase portraits of system (27) for *c* = 0 and for the same positive values of *k* as those in Figure 4. Red points are equilibrium points. Green curves are nullclines and blue curves are trajectories.

Phase portraits of system (27) for *c* = 0 and for the same positive values of *k* as those in Figure 4 can be found in Figure 5.

#### 3.3.2. For *c* > 0

Let us introduce the following notation:

(31) $$\begin{array}{c} M_{k}\equiv \max\left\{\;k\phi +2\sqrt{D^{\prime }\left(\phi \right)g\left(\phi \right)}\;\right\}, \end{array}$$

where the maximum is taken on the closed interval [0,1]. Through this subsection we are going to distinguish two main cases:

(1) 0 < *c* < *M*_*k*_,
(2) *c* ≥ *M*_*k*_.

For values of *c* satisfying *c* ≥ *M*_*k*_, *V*_1_ and *V*_2_ are defined1 for all *ϕ* ≥ 0; in particular they do so on the interval [0,1].

**Figure 6:**
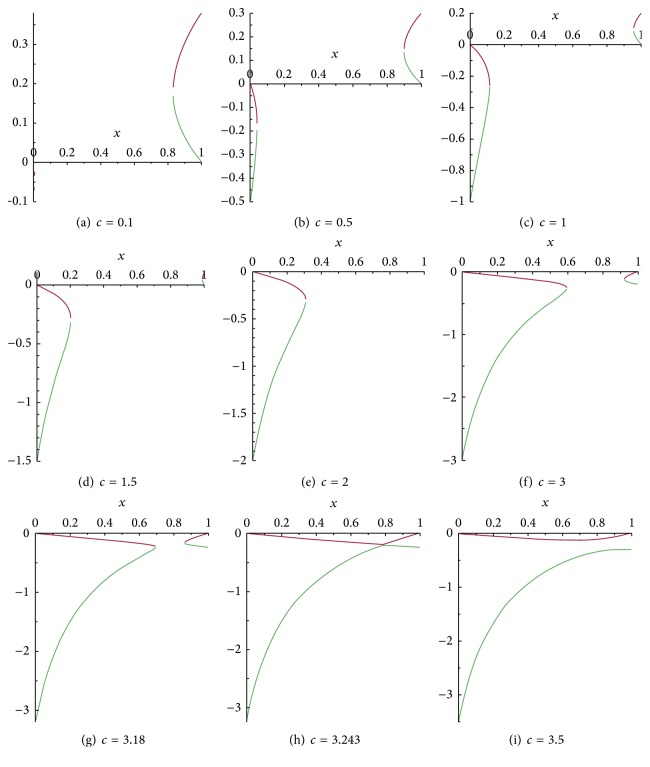
Behaviour of the two branches of the vertical nullclines of system (18) for fixed *k* = 2 and *c* > 0: (a)–(g) for 0 < *c* < *M*_*k*_, (h) for *c* = *M*_*k*_, and (i) for *c* > *M*_*k*_. Here *β* = 2.

In another side, for values of *c* such that 0 < *c* < *M*_*k*_ these branches of the vertical nullcline are not defined on the whole interval [0,1] but they do so on the union of subintervals contained within it.  Figure 6 illustrates the behavior of (25) in the same representative2 case as in previous subsection for fixed positive *β* and *k*. Here, the positive *c* varies.

Now, we use this behavior to determine the phase portrait of (18). We proceed by considering extreme values of *c*. We can prove the following proposition.

**Proposition 6 .**

For each value of *c* such that *c* ≥ *M*_*k*_ system (18) has a heteroclinic trajectory (*ϕ*_*c*_(*τ*), *v*_*c*_(*τ*)), connecting the equilibria *P*_1_ and *P*_0_, that is, satisfying

(1) 0 < *ϕ*_*c*_(*τ*) < 1 and *v*_*c*_(*τ*) < 0 for all *τ* ∈ (−*∞*, +*∞*),
(2) lim_*τ*→−*∞*_⁡(*ϕ*_*c*_(*τ*)  , *v*_*c*_(*τ*)) = (1,0) and lim_*τ*→+*∞*_⁡(*ϕ*_*c*_(*τ*), *v*_*c*_(*τ*)) = (0,0).

**Proof**

For *c* ≥ *M*_*k*_ the vertical null-clines look like in Figures 6(h) or 6(i). On each one of these branches the vector field, being horizontal, points out towards the left; meanwhile on the region {(*ϕ*, *v*)∣0 < *ϕ* < 1, *V*_2_(*ϕ*) < *v* < *V*_1_(*ϕ*)}, the vector field points left up. This behavior allows us to construct a positive invariant region for such a vector field in a similar fashion as that carried out in [25]. In fact, we can select a function *f* : [0,1] → *ℝ* belonging to the set *C*_[0,1]_^1^ satisfying (a) *f*(0) = *v*_0_ < 0, *f*(1) = 0; (b) *f*′(*ϕ*) > 0  ∀*ϕ* ∈ (0,1); and *V*_2_(*ϕ*) < *f*(*ϕ*) < *V*_1_(*ϕ*) in such a way the restriction of the vector field (18) on the graph of *f* points inwards the region {(*ϕ*, *v*)∣0 < *ϕ* < 1, *f*(*ϕ*) < *v* < 0}. Then, by a straightforward application of the Poincaré-Bendixson Theorem we have that any trajectory of (18)—in particular that leaving *P*_1_ following the left branch of the unstable manifold at *P*_1_—entering this region, must end at one equilibrium point. Given that there is not any other possibility such trajectory must end at *P*_0_. Hence the proof follows.

**Figure 7:**
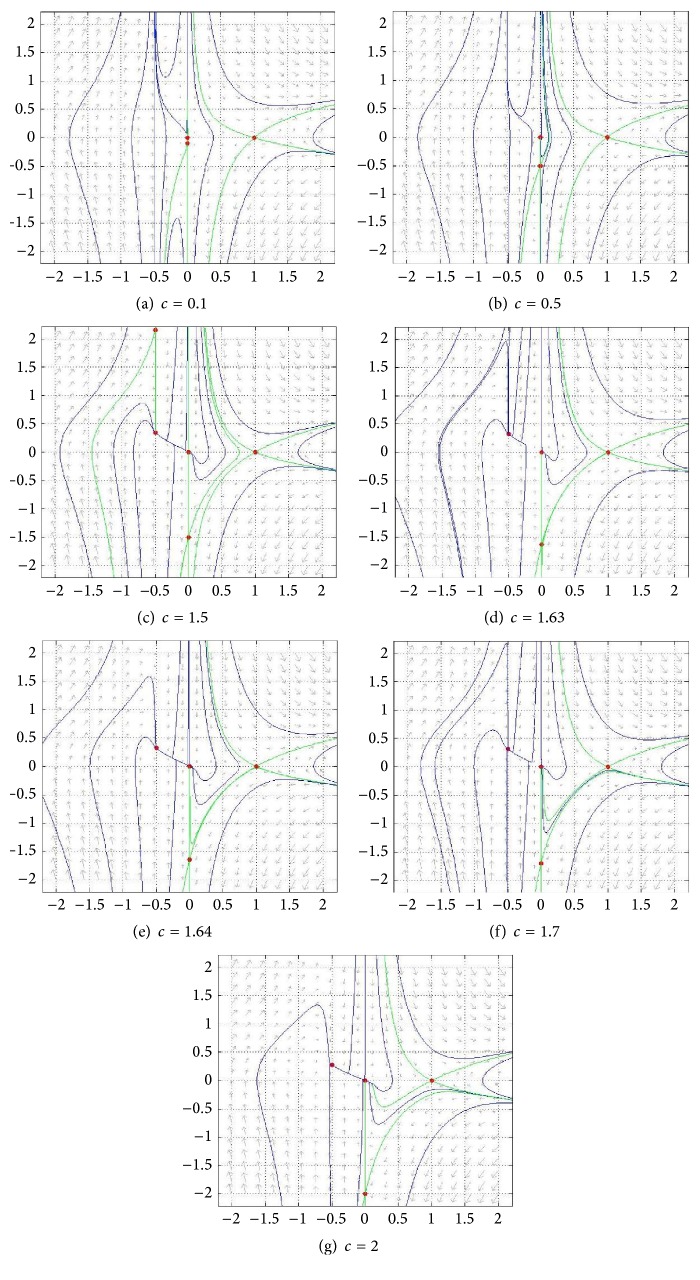
Phase portrait of (18) for fixed *k* = 2 and *c* > 0. In (a)–(c) there are no heteroclinic trajectories. (d) shows the existence of a saddle-saddle heteroclinic trajectory for *c* ^*∗*^ ≈ 1.63. (e)–(g), for the indicated value of *c*, show a saddle (*P*_1_) saddle-node (*P*_0_) connection. Here *β* = 2. Red points are equilibrium points, green curves are nullclines, and blue curves are trajectories.

The phase portrait of (18) for fixed *k* = 2 and *c* > 0 can be seen in Figure 7.

### 3.4. A Monotonicity Property

Let (*ϕ*, *v*) be any fixed (but arbitrary) point belonging to the region

(32) $$\begin{array}{c} F_{1}=\left\{\;\left(\;\phi ,v\;\right)\;|\;0<\phi <1,\;\;-\infty <v<0\;\right\}. \end{array}$$

For each pair (*c*, *k*), let us denote by *θ*(*ϕ*, *v*; *c*, *k*) the angle formed by the vector field (18) with the positive semihorizontal *ϕ*-axis. Then

(33) $$\begin{array}{c} \tan\theta \left(\;\phi ,v;c,k\;\right)=\frac{-\left(c-k\phi \right)v-D^{\prime }\left(\phi \right)v^{2}-g\left(\phi \right)}{D\left(\phi \right)v}. \end{array}$$

**Proposition 7 .**

The angle *θ*(*ϕ*, *v*; *c*, *k*) is a monotone decreasing function of the parameter *c* and a monotone increasing function with respect to the parameter *k*.

**Proof**

Calculating the partial derivative with respect to *c* in the equality

(34) θϕ,v;c,k=tan−1⁡−c−kϕv−D′ϕv2−gϕDϕv,

we obtain

(35) ∂θ∂cϕ,v;c,k=−Dϕv2Dϕv2+−c−kϕv−D′ϕv2−gϕ2<0;

meanwhile the corresponding partial derivative with respect to *k* is

(36) ∂θ∂kϕ,v;c,k=−kϕDϕvDϕv2+−c−kϕv−D′ϕv2−gϕ2>0,∀ϕ,v∈F1.

Then the proof follows.

Both monotonicity properties contained in the above proposition have important implications in refining the analysis for searching heteroclinic trajectories of system (18) connecting *P*_1_ with *P*_*c*_ or with *P*_0_. In particular, for fixed *k* > 0, if we continuously decrease the parameter *c* starting from *M*_*k*_, the left branch of the unstable manifold at *P*_1_, *W*_*c*_^*u*^(*P*_1_), will move continuously downwards within the region *ℱ*_1_; meanwhile, the right branch of the stable manifold at *P*_*c*_, *W*_*c*_^*s*^(*P*_*c*_), moves continuously upwards as *c* decreases in the same region. By continuity of the vector field with respect to the parameter *c* and using shooting arguments, there exists a unique, *c* ^*∗*^ > 0, value of *c* for which both manifolds touch each other resulting in a saddle-saddle heteroclinic trajectory connecting the equilibrium points *P*_1_ with *P*_*c*^*∗*^_. This reasoning constitutes the proof of the following lemma.

**Lemma 8 .**

For the functions *D*(*u*) = *u* + *βu* ^2^ and *g*(*u*) = *u*(1 − *u*) with *β* > 0 and for *h*′(*u*) = *ku* with *k* > 0 there exists a unique *c* ^*∗*^—depending on *k*—positive value of *c* for which system (18) has a unique heteroclinic trajectory connecting the equilibria *P*_1_ and *P*_*c*_. Moreover

(1) By increasing any of the parameters *c* or *k*, such a trajectory is destroyed and for each (*c*, *k*) with either *c* > *c* ^*∗*^ or *k* > *k* ^*∗*^ a heteroclinic trajectory connecting *P*_1_ with *P*_0_ emerges.
(2) On the contrary, by decreasing any of these parameters there are not heteroclinic trajectories for system (18) at all.

**Proof**

This follows from Propositions 4–7.

Note that there are two reasons why system (18) is not structurally stable (see Peixoto's theorem in [24]). These are the existence of a nonhyperbolic point and a saddle-saddle heteroclinic trajectory. In particular, any small perturbation of such system, for example, by varying the parameter *c* in a small neighborhood, *V*_*ε*_(*c* ^*∗*^), of the critical value *c* ^*∗*^, involves strong dynamical changes including the destruction of the saddle-saddle trajectory and the emergence of a saddle (*P*_1_) saddle-node (*P*_0_) connexion or the disappearance of heteroclinic trajectories at all.

**Theorem 9 .**

For the functions *D*(*u*) = *u* + *βu* ^2^ and *g*(*u*) = *u*(1 − *u*) with *β* > 0 and for *h*′(*u*) = *ku* with *k* > 0, given *k* there exists a unique critical value, *c* ^*∗*^ (depending on *k*), of *c* such that (14) has

(1) no traveling wave solutions for *c* such that 0 < *c* < *c* ^*∗*^,
(2) a unique traveling wave solution of sharp type for *c* = *c* ^*∗*^,
(3) a monotonic decreasing traveling wave solution for each *c* such that *c* > *c* ^*∗*^.

**Proof**

The previous analysis demonstrates the existence of the associated heteroclinic trajectories (see Lemma 8). Searching for travelling wave solutions of the PDE (3), given that such solutions have the particular form *u*(*x*, *t*) = *ϕ*(*x* − *ct*) ≡ *ϕ*(*ξ*), is equivalent to showing the existence of heteroclinic trajectories of the associated ODE system (18). Therefore the theorem follows.

**Figure 8:**
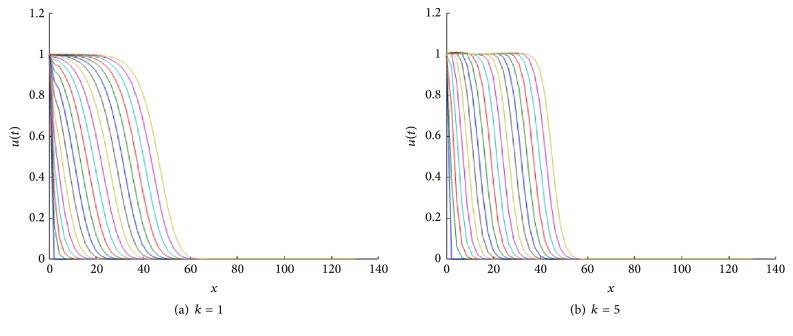
Front traveling waves solutions of (14) at equal time intervals *t* = 1,2, 3,… with *g*(*u*) = *u*(1 − *u*) and *D*(*u*) = *u*(1.0 + *βu*) for *β* = 1. The values for *k* are indicated in each figure.

**Figure 9:**
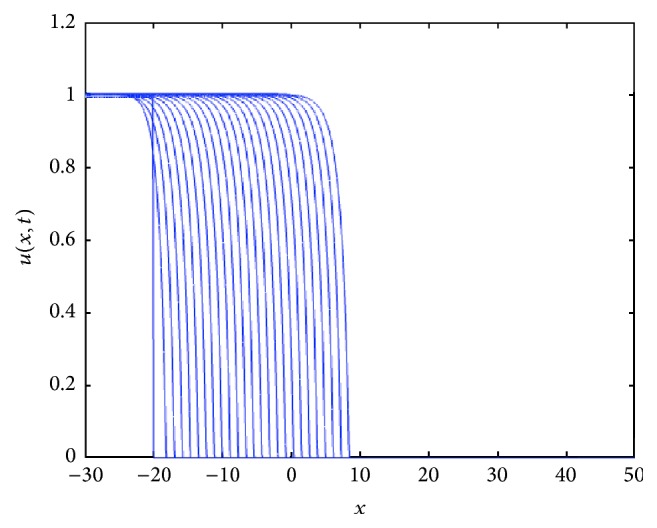
Evolution of a compact support function as initial condition under the reaction-diffusion convection process described by (14). The numerics show that as the time grows, the approximative solution tends to the sharp traveling wave solution. Profiles are plotted at equal time intervals *t* = 1,2, 3,… with *g*(*u*) = *u*(1 − *u*) and *D*(*u*) = *u*(1.0 + *βu*) for *β* = 1 and *h*′(*u*) = *ku*, with *k* = 1.

In Figure 8 we show two front traveling wave profiles for two values of *k*. In Figure 9 the sharp traveling wave can be seen.

We close this section by numerically exploring the influence of changes in *k* on the critical value, *c* ^*∗*^, of *c* for which the r-d-a equation has a sharp type solution. This is the content of the next subsection.

### 3.5. The Speed *c* ^*∗*^ Depending on *k* in a Particular Case

As we already mentioned in Section 1, the equation *u*_*t*_ = *u*_*xx*_ − *kuu*_*x*_ + *u*(1 − *u*) has a monotone decreasing TWS connecting the states *u*_1_(*x*, *t*) ≡ 1 and *u*_0_(*x*, *t*) ≡ 0 for each *c* ≥ *c*(*k*), where the explicit form of *c*(*k*) is given by (1). This result by Murray was our motivation for seeking the corresponding relationship between the speed for which our reaction-diffusion-convection equation (14) has TWS and the parameter *k*, in particular for those of sharp type.

**Figure 10:**
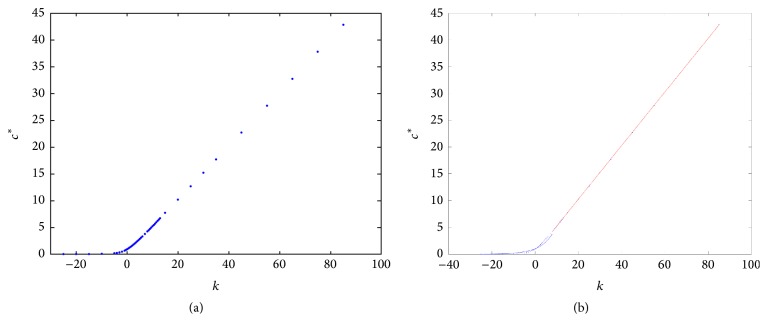
“Numerical experimental data” for *c* ^*∗*^ for different values of *k* with *h*′(*u*) = *ku*. The data as they were obtained (a); the fitting (b).

In this subsection we illustrate this relationship through a particular case. To this aim, we choose *D*(*u*) = *u* + *βu* ^2^ and *g*(*u*) = *u*(1 − *u*) with *β* > 0. Through this subsection our approach is from a numerical point of view. We carried numerical simulations of the phase portrait of the corresponding nonsingular ODE system in the traveling wave coordinate. The goal of this was to illustrate, for different values of *k*, the corresponding critical values, *c* ^*∗*^, of *c* for which a unique saddle-saddle heteroclinic trajectory exists (one for each *k*). As we already know, associated with this trajectory, there exists a unique TWS of sharp type for (6). In Figure 10(a) we present a numerical approximation of how *c* ^*∗*^ depends on *k* through the corresponding phase portrait, with *D*(*u*) and *g*(*u*) as before.

This information tells us that, on this range of the “numerical experiments,” the speed *c* ^*∗*^ is a growing function of *k*. Moreover, we can distinguish two qualitative parts: one exponential for *k* ≤ 8 and another linear for *k* ≥ 8. We carried out the corresponding fittings. These are our results for each phase:

(37) c∗k=1.007151763e0.1662829302·k,for  k≤8,

(38) c∗k=0.5010815735·k+0.2125604852,for  k≥8,

respectively. See Figure 10(b).

**Figure 11:**
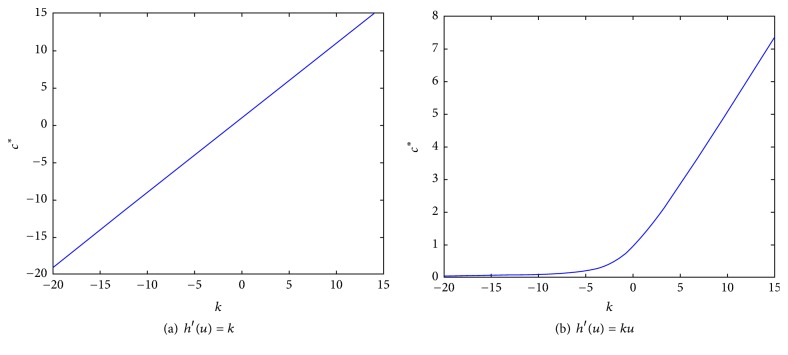
Plot *c* ^*∗*^ as function of the parameter *k*: for *h*′(*u*) = *k* and *h*′(*u*) = *ku*.

In Figure 11 we show how *c* ^*∗*^ changes as function of the parameter *k* for *h*′(*u*) = *k* and *h*′(*u*) = *ku* for comparison.

As result of the numerical experiments, we can see that for *k* < 0 the critical value *c* ^*∗*^ is small but positive, but once *k* increases, the values of *c* ^*∗*^ increase faster with *k*. The physical interpretation of this is as follows: for *k* < 0 the “wind” *h*′(*u*) = *k* acts in the opposite direction to which the wave travels; meanwhile for *k* > 0, the advective term pushes in the same direction as the traveling wave goes. As a result of this, the critical values of the speed, for which there exists the sharp wave, increase as *k* increases.

## 4. Traveling Wave Solutions Analysis in the General RDA Equation

The particular cases discussed in previous sections give us some insights in order to carry out the TWS analysis for the general RDA equation:

(39) ∂u∂t=∂∂xDu∂u∂x−h′u∂u∂x+gu,∀x,t∈R×R+,

where *D*, *g*, and *h* are real functions defined on the interval [0,1]. The first two functions satisfy conditions (1) and (3) stated at the beginning of Section 1; meanwhile *h*, in addition to satisfying the conditions in item (2), is such that *h*′(0) might have different signs. In particular when *h*′(0) = 0 (39) is degenerate in both the diffusion and the advective terms.

For the analysis of TWS we proceed in a standard way: let us assume *u*(*x*, *t*) = *ϕ*(*x* − *ct*) ≡ *ϕ*(*ξ*) with *c* > 0 is solution of the RDA equation (39). Thus, by substituting *ϕ* in (39) we obtain a nonlinear second-order ODE equation for *ϕ* which, by introducing *v* = *ϕ*′(*ξ*), can written as a singular (at *ϕ* = 0) nonlinear ODE system. The singularity can be removed by introducing the parameter *τ* in a similar fashion as we did in previous sections (see [19, 26]). The result is the following nonsingular and nonlinear ODE system:

(40a) ϕ˙=Dϕv≡f1ϕ,v,

(40b) v˙=−c−h′ϕv−D′ϕv2−gϕ≡f2ϕ,v,

where the dot on *ϕ* and *v* denotes the derivative with respect to *τ*. This system and the singular system are topologically equivalent on the stripe

(41) $$\begin{array}{c} F=\left\{\;\left(\;\phi ,v\;\right)\;|\;0<\phi <1,\;-\infty <v<\infty \;\right\}. \end{array}$$

The analysis of the system ((40a) and (40b)) starts by obtaining its nullclines. The horizontal nullcline is the coordinate axis of the *ϕv* plane. The vertical nullcline has the following two branches:

(42) V1ϕ=−c−h′ϕ+c−h′ϕ2−4D′ϕgϕ2D′ϕ,V2ϕ=−c−h′ϕ−c−h′ϕ2−4D′ϕgϕ2D′ϕ.

Given the conditions on *D*, *h*, and *g*, the functions *V*_1_ and *V*_2_ are defined on the whole interval [0,1] whenever the inequality,

(43) $$\begin{array}{c} {\left[\;c-h^{\prime }\left(\;\phi \;\right)\;\right]}^{2}\geq 4D^{\prime }\left(\;\phi \;\right)g\left(\;\phi \;\right),\;\forall \phi \in \left[0,1\right], \end{array}$$

holds which, in turn, gives us a bound for *c* for which the two branches (42) are defined on [0,1]. This is

(44) $$\begin{array}{c} c\geq h^{\prime }\left(\;\phi \;\right)+2\sqrt{D^{\prime }\left(\phi \right)g\left(\phi \right)}. \end{array}$$

When the above inequality does not hold, *V*_1_ and *V*_2_ are not defined on the whole interval [0,1]. In fact, they are defined on disjoint intervals belonging to the interval [0,1].

From the explicit form of *V*_1_ and *V*_2_ it follows *V*_1_(0) = 0, *V*_1_(1) = 0, and

(45) V20=−c−h′0D′0,V21=−c−h′1D′1.

Since we assumed both *D*′(0) and *D*′(1) are positive, depending on *c* compared with *h*′(0) (or with *h*′(1)), the following cases might occur:

(i) *V*_2_(0) < 0 for *c* > *h*′(0), *V*_2_(1) < 0 for *c* > *h*′(1),
(ii) *V*_2_(0) = 0 for *c* = *h*′(0), *V*_2_(1) = 0 for *c* = *h*′(1),
(iii) *V*_2_(0) > 0 for *c* < *h*′(0), *V*_2_(1) > 0 for *c* < *h*′(1).

The equilibrium points of (40a) and (40b) are

(46) P0=0,0,P1=1,0,Pc,h′0=0,−c−h′0D′0=0,V20.

Given the positiveness of *D*′(0), depending on the sign of (*h*′(0) − *c*) the third equilibrium is located on the

(i) positive vertical *v*-semiaxis, for *c* < *h*′(0),
(ii) the origin for *c* = *h*′(0),
(iii) negative vertical *v*-semiaxis, for *c* > *h*′(0).

### 4.1. Local Dynamics

The linear local analysis of the system ((40a) and (40b)) is based on the Jacobian matrix, *J*[*f*_1_, *f*_2_], of the vector field (*f*_1_, *f*_2_) evaluated at the equilibria. The Jacobian matrix at any point (*ϕ*, *v*) is

(47) Jf1,f2ϕ,v=D′ϕvDϕh′′ϕ−D′′ϕvv−g′ϕ−c−h′ϕ−2D′ϕv.

At *P*_0_, det⁡*J*[*f*_1_, *f*_2_]_(0,0)_ = 0 and tr⁡*J*[*f*_1_, *f*_2_]_(0,0)_ = −(*c* − *h*′(0)). Hence, whenever *c* ≠ *h*′(0), *P*_0_ is a nonhyperbolic point of codimension one. Evaluating *J*[*f*_1_, *f*_2_]_(*ϕ*,*v*)_ at *P*_1_ we obtain det⁡*J*[*f*_1_, *f*_2_]_(1,0)_ = *g*′(1)*D*(1) < 0 and tr⁡*J*[*f*_1_, *f*_2_]_(1,0)_ = −(*c* − *h*′(1)); hence *P*_1_ is a hyperbolic saddle point. Finally, the evaluation of *J*[*f*_1_, *f*_2_] at *P*_(*c*,*h*′(0))_ gives us tr⁡*J*[*f*_1_, *f*_2_]_*P*_(*c*,*h*′(0))__ = 0 and det⁡*J*[*f*_1_, *f*_2_]_*P*_(*c*,*h*′(0))__ = −(*c* − *h*′(0))^2^ from which it follows that for *c* ≠ *h*′(0), det⁡*J*[*f*_1_, *f*_2_]_*P*_(*c*,*h*′(0))__ < 0. Thus, whenever *c* ≠ *h*′(0), *P*_(*c*,*h*′(0))_ is a hyperbolic saddle. On the contrary, for *c* = *h*′(0) the equilibrium *P*_(*c*,*h*′(0))_ is a nonhyperbolic point of codimension two.

The Jacobian at *P*_0_ is

(48) $$\begin{array}{c} J{\left[\;f_{1},f_{2}\;\right]}_{\left(0,0\right)}=\left(\begin{array}{cc} 0 & 0\\ -g^{\prime }\left(0\right) & h^{\prime }\left(0\right)-c \end{array}\right) \end{array}$$

with eigenvalues *λ*_1_(*P*_0_) = 0, *λ*_2_(*P*_0_) = *h*′(0) − *c* and eigenvectors **v**_1_ = (−(*c* − *h*′(0))/*g*′(0), 1), **v**_2_ = (0,1), respectively.

The Jacobian at *P*_1_ is

(49) $$\begin{array}{c} J{\left[\;f_{1},f_{2}\;\right]}_{\left(1,0\right)}\left(\begin{array}{cc} 0 & f\left(1\right)\\ -g^{\prime }\left(1\right) & h^{\prime }\left(1\right)-c \end{array}\right) \end{array}$$

with eigenvalues $\lambda_{1}(P_{1})=\left(1/2\right)\left(-c+h^{\prime }(1)-\sqrt{{\left(c-h^{\prime }(1)\right)}^{2}-4D\left(1\right)g^{\prime }(1)}\right)$,  $\lambda_{2}(P_{1})=\left(1/2\right)\left(-c+h^{\prime }(1)+\sqrt{{\left(c-h^{\prime }(1)\right)}^{2}-4D\left(1\right)g^{\prime }(1)}\right)$ and eigenvectors **v**_1_ and **v**_2_ given by

(50) −c−h′1−c2−2h′1c+h′12−4D1g′12g′1,1,−c−h′1+c2−2h′1c+h′12−4D1g′12g′1,1,

respectively.

The Jacobian at *P*_(*c*,*h*′(0))_ is

(51) $$\begin{array}{c} J{\left[\;f_{1},f_{2}\;\right]}_{\left(0,-\left(c-h^{\prime }\left(0\right)\right)/D^{\prime }\left(0\right)\right)}\left(\begin{array}{cc} h^{\prime }\left(0\right)-c & 0\\ -\frac{D^{\prime \prime }\left(0\right){\left(h^{\prime }\left(0\right)-c\right)}^{2}}{D^{\prime }{\left(0\right)}^{2}}+\frac{h^{\prime \prime }\left(0\right)\left(h^{\prime }\left(0\right)-c\right)}{D^{\prime }\left(0\right)}-g^{\prime }\left(0\right) & -c-2\left(h^{\prime }\left(0\right)-c\right)+h^{\prime }\left(0\right) \end{array}\right) \end{array}$$

with eigenvalues *λ*_1_(*P*_(*c*, *h*′(0))_) = *h*′(0) − *c*, *λ*_2_(*P*_(*c*, *h*′(0))_) = *c* − *h*′(0) and eigenvectors

(52) v1=2D′02c−h′0g′0D′02+c−h′0h′′0D′0+c−h′02D′′0,1,v2=0,1,

respectively.

### 4.2. Degeneracy Just in the Diffusion Term at *u* = 0

Here *D*(0) = 0 and *h*′(0) ≠ 0. Thus, for a fixed *k* and positive *c*—as we assumed—depending on the sign of *h*′(0) its effect on *P*_(*c*,*h*′(0))_ is as follows:

(1) For *h*′(0) > 0 we have two subcases:
  (a) 0 < *h*′(0) < *c*; here *P*_(*c*,*h*′(0))_ is closer to *P*_0_ than *P*_*c*_ does; this is on the negative vertical *v*-axis.
  (b) 0 < *c* < *h*′(0); *P*_(*c*,*h*′(0))_ switches to the positive vertical axis of the *ϕv* plane.
(2) For *h*′(0) < 0 the equilibrium runs away on the vertical negative axis. Here *P*_(*c*,*h*′(0))_, for all *c* > 0, is more far away from *P*_0_ on the vertical negative axis.

#### 4.2.1. *h*′(0) > 0

**Figure 12:**
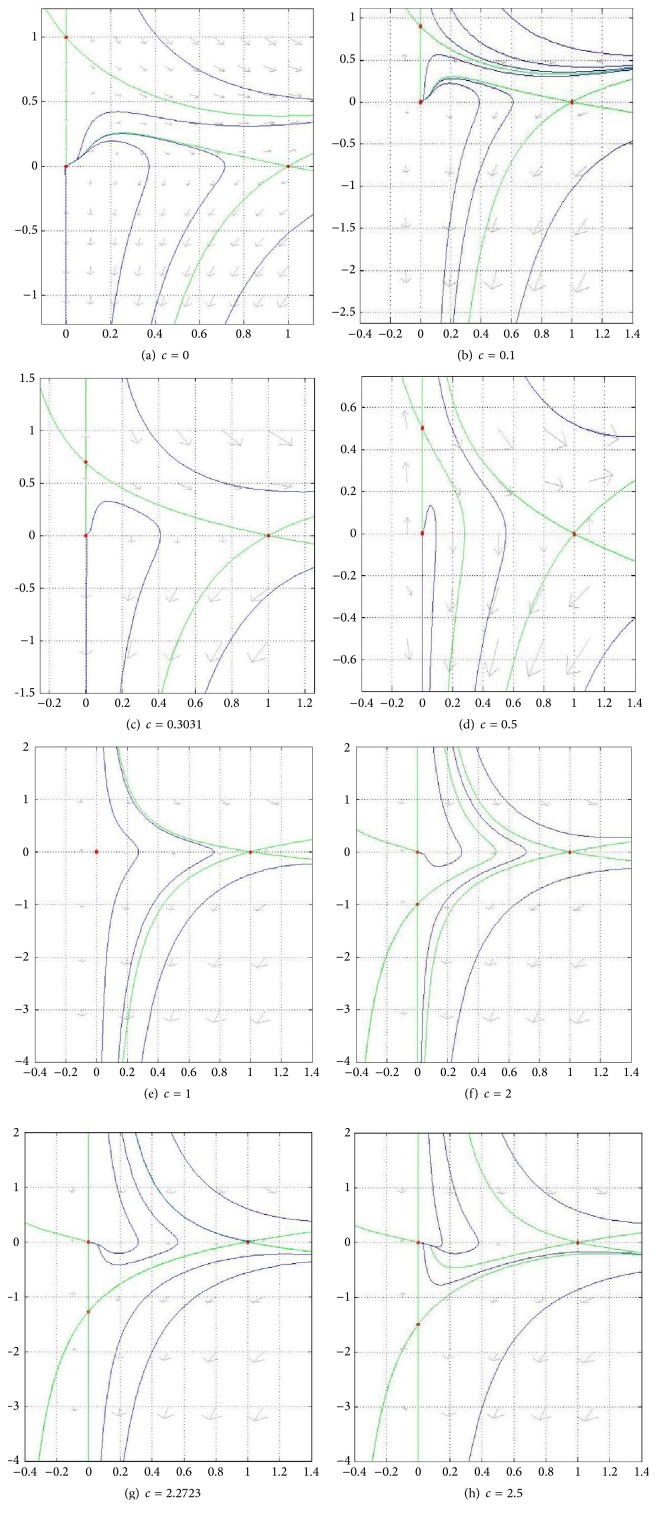
Phase portrait of (40a) and (40b) for eight values of *c*; (a)–(h) contemplate the first subcase (1 = *h*′(0) > *c*); (e) shows *c* = 1 (only two equilibria); and (f)–(h) contain the second subcase (*c* > *h*′(0) = 1). Here *k* = *γ* = *α* = 1, *β* = 2. Red points are equilibrium points. Green curves are nullclines and blue curves are trajectories.

We will take *h*′(*u*) = *α* + *γu* (*α* > 0) as an example of the first case. The behavior of the phase portrait depends on how *h*′(0) compares with *c*. In this example this means how *α* compares to *c*. For our example *h*′(0) = *α* = 1, we show in Figure 12 the phase portrait for several values of *c*. In particular, as stated before, we have two major subcases: 0 < *c* = 1 < *h*′(0) and 0 < *h*′(0) = 1 < *c*. For completeness, we start by showing the phase portrait for *c* = 0. In Figures 12(a) and 12(b) there are three equilibrium points and a heteroclinic connexion from *P*_0_ to *P*_1_, in Figure 12(c) the heteroclinic connexion is from *P*_*c*_ to *P*_1_, in Figure 12(d) there are only trivial heteroclinic connexions, in Figure 12(e) there are only two equilibria, in Figure 12(f) there are only trivial connexions, Figure 12(g) corresponds to *c* ^*∗*^, that is, one connexion from *P*_1_ to *P*_*c*_, and in Figure 12(h) for each *c* > *c* ^*∗*^ there is connexion from *P*_1_ to *P*_0_.

**Figure 13:**
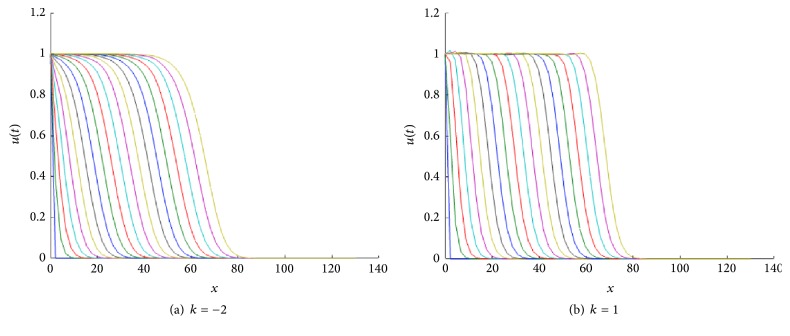
Front traveling waves solutions of (39) for *h*′(*u*) = *α* + *γu* at equal time intervals *t* = 1,2, 3,… with *g*(*u*) = *u*(1 − *u*) and *D*(*u*) = *u*(1.0 + *βu*) for *β* = 1. The values for *k* are indicated in each figure.

**Figure 14:**
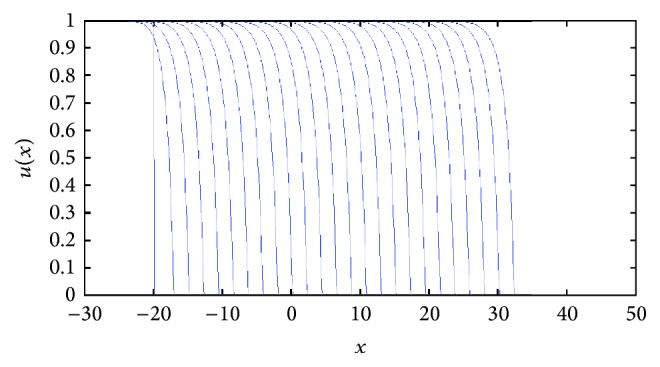
Evolution of a compact support function as initial condition under the reaction-diffusion convection process described by (39). The numerics show that as the time grows, the approximative solution tends to the sharp traveling wave solution. Profiles are plotted for *h*′(*u*) = *α* + *γu* at equal time intervals *t* = 1,2, 3,… with *g*(*u*) = *u*(1 − *u*) and *D*(*u*) = *u*(1.0 + *βu*) for *β* = *k* = 1.

**Figure 15:**
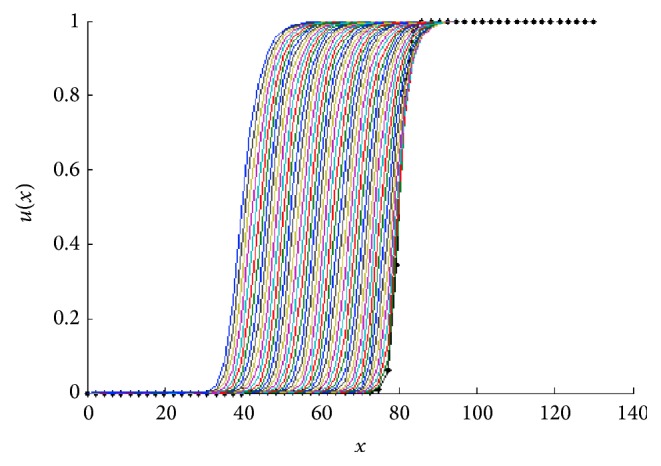
Front traveling waves solutions of (39) for *h*′(*u*) = *α* + *γu* at equal time intervals *t* = 1,2, 3,… with *g*(*u*) = *u*(1 − *u*) and *D*(*u*) = *u*(1.0 + *βu*) for *β* = *k* = 1.

**Figure 16:**
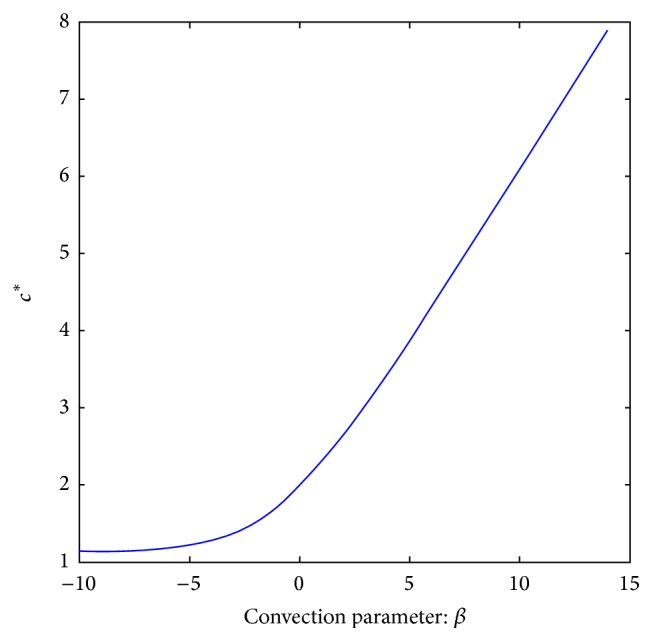
Plot of how *c* ^*∗*^ changes with *k* for *h*′(*u*) = *α* + *γu*. For a comparison, refer to Figure 10, where *h*′(*u*) = *ku*.

In Figure 13 we show the corresponding front traveling wave profiles for two values of *k*. These were obtained by numerically solving (39) for *h*′(*u*) = *α* + *γu* with the numerical routine NAG P03PCF (NAG Matlab Toolbox).  Figure 14 shows the sharp traveling wave. We also plot how *c* ^*∗*^ changes with *k* in this case (see Figure 16). Although we are concentrating on heteroclinic connections leaving *P*_1_, we display the phase portrait behavior for a broad range of values for *k* to show the richness of the dynamics. To this end, we would also like to show how the heteroclinic connexion from *P*_0_ to *P*_1_ from Figure 12(b) translates into a front traveling from right to left (the initial condition in that case is marked with dots) connecting 0 with 1; see Figure 15.

#### 4.2.2. *h*′(0) < 0

**Figure 17:**
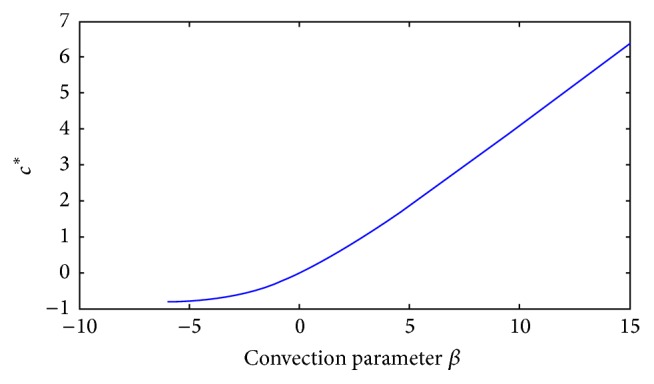
Plot of how *c* ^*∗*^ changes with *k* for *h*′(*u*) = *α* + *γu*, with *α* = −1 < 0. For a comparison, refer to Figure 10, where *h*′(*u*) = *ku*.

This case is basically the same as before. We have two main subcases according to the sign of *c* − *h*′(0).  Figure 17 shows how *c* ^*∗*^ changes with *k* for *h*′(*u*) = *α* + *γu*. We do not plot the corresponding phase portraits or traveling waves as they are qualitatively similar to the ones in the previous subsection. We do, however, show how the advection parameter changes with *k* in Figure 17; note how here also there is linear dependency to later become an exponential one. For a comparison, refer to Figure 10.

### 4.3. Degeneracy in Both Diffusion and Advection Terms at *u* = 0

Here *D*(0) = *h*′(0) = 0. Then the equilibrium *P*_(*c*,0)_ coincides with the equilibrium, *P*_*c*_, which is allocated on the seminegative vertical *v*-axis for the corresponding system when no advection term is included. Here we will take *h*′(*u*) = *u* + *ku* ^2^ as an example.

*Mutatis mutandis* one of the monotonicity properties contained in Proposition 7 holds for the general case. In fact, by using the same notation as that used there, the following proposition holds.

**Proposition 10 .**

For fixed (but arbitrary) (*ϕ*, *v*) ∈ *ℱ*_1_ the angle, *θ*(*ϕ*, *v*; *c*), formed by the positive *ϕ* semihorizontal axis and the vector field which defines the system ((40a) and (40b)), is a monotone decreasing function of the parameter *c*.

**Proof**

We have

(53) θϕ,v;c=tan−1⁡−c−h′ϕv−D′ϕv2−gϕDϕv,

and then, by simply calculating the derivative with respect to *c* in the above equality, we obtain

(54) ∂θ∂cϕ,v;c=−Dϕv2Dϕv2+−c−h′ϕv−D′ϕv2−gϕ2<0,

for all (*ϕ*, *v*) ∈ *ℱ*_1_; then the proof follows.

Of course, all the implications this proposition has are assumed by the corresponding ODE system which in turn implies the existence of sharp and monotone traveling wave solutions.

**Figure 18:**
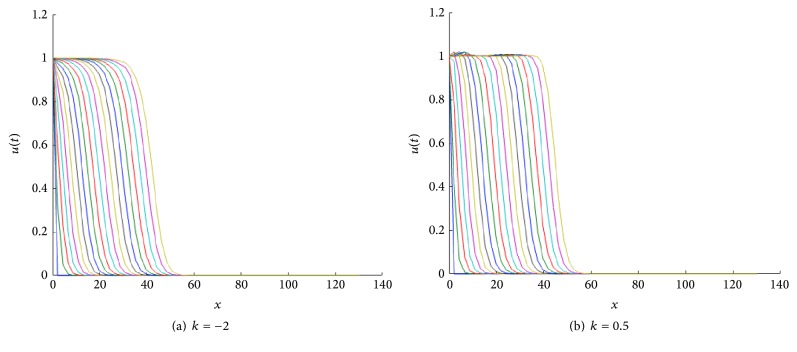
Front traveling waves solutions of (39) for *h*′(*u*) = *u* + *ku* ^2^ at equal time intervals *t* = 1,2, 3,… with *g*(*u*) = *u*(1 − *u*) and *D*(*u*) = *u*(1.0 + *βu*) for *β* = 1. The values for *k* are indicated in each figure.

**Figure 19:**
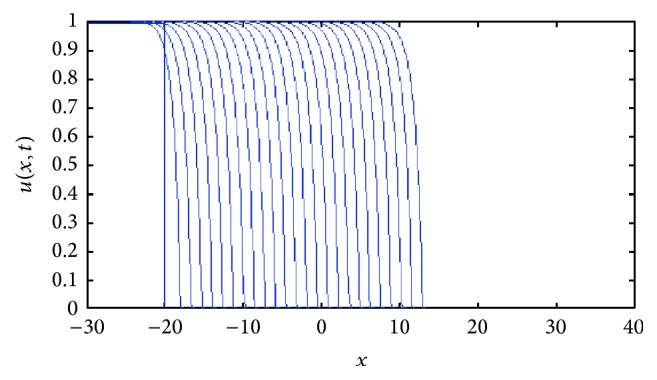
Evolution of a compact support function as initial condition under the reaction-diffusion convection process described by (39). The numerics show that as the time grows, the approximative solution tends to the sharp traveling wave solution. Profiles are for *h*′(*u*) = *u* + *ku* ^2^ at equal time intervals *t* = 1,2, 3,… with *g*(*u*) = *u*(1 − *u*) and *D*(*u*) = *u*(1.0 + *βu*) for *β* = 1.

In Figure 18 we show the corresponding front traveling wave profiles for two values of *k* for *h*′(*u*) = *u* + *ku* ^2^ and in Figure 19 the sharp type.

**Figure 20:**
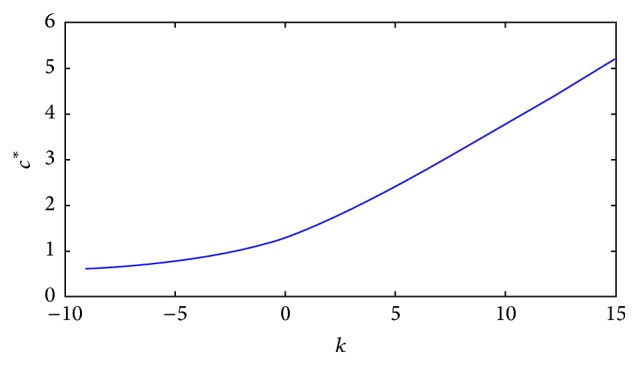
Plot of how *c* ^*∗*^ changes with *k* for *h*′(*u*) = *u* + *ku* ^2^. For a comparison, refer to Figure 10, where *h*′(*u*) = *ku*.

Figure 20 shows how *c* ^*∗*^ changes with *k* for *h*′(*u*) = *u* + *ku* ^2^.

## 5. Discussion

We have presented a study on the effect of the incorporation of a nonlinear advection term in the degenerate reaction-diffusion equation (3). When investigating the traveling wave behavior, we found that the “advection speed” influences the type and the speed of the possible traveling waves. The aim of this paper was the investigation of the existence of TWS for the one-dimensional nonlinear degenerate RDA equation (3). The degeneracy of the equation causes its solution to possess finite speed of propagation throughout the space.

The TWS analysis for (3) was carried out for three cases: (1)  *h*′(*u*) = *k*, (2)  *h*′(*u*) = *ku*, and (3) no specific form for *h*′(*u*), as long as it satisfies the quite basic requirements as stated in item (2). In all these cases, (3) has

(1) no traveling wave solutions for 0 < *c* < *c* ^*∗*^,
(2) a unique traveling wave solution of sharp type for *c* = *c* ^*∗*^,
(3) a traveling wave solution of smooth decreasing front type satisfying the boundary conditions lim_*ξ*→−*∞*_⁡*ϕ*(*ξ*) = 1 and lim_*ξ*→+*∞*_⁡*ϕ*(*ξ*) = 0 with 0 < *ϕ*(*ξ*) < 1  ∀*ξ* ∈ (−*∞*, +*∞*), for each *c* > *c* ^*∗*^.

We also numerically solved the initial and boundary value problems associated with the full RDA in each considered case. Moreover, we show how the advection speed impacts the type and speed of the traveling waves. In particular, the unique *c* = *c* ^*∗*^ for which there are sharp traveling waves depends initially linearly on the advection speed to later increase exponentially.

## Endnotes

1. Note that, certainly, the inequality *c* ≥ *M*_*k*_ gives us a bound of *c* for which both branches are defined on [0,1]. However, for *c* satisfying $c\geq k\phi +2\sqrt{D^{\prime }(\phi )g(\phi )}$ it is enough for the definitiveness of *V*_1_ and *V*_2_ on the interval [0,1].

2. Note that for the representative case *D*(*ϕ*) = *ϕ* + *βϕ* ^2^ and *g*(*ϕ*) = *ϕ*(1 − *ϕ*) with *β* > 0, the corresponding ODE system (18) could have another two equilibria. These are $\left(\tilde{\phi },{\tilde{v}}_{1}\right)$ and $\left(\tilde{\phi },{\tilde{v}}_{2}\right)$, where

(∗) $$\begin{array}{c} {\tilde{v}}_{1,2}=\frac{-\left(c-k\tilde{\phi }\right)\pm \sqrt{{\left(c-k\tilde{\phi }\right)}^{2}-4D^{\prime }\left(\tilde{\phi }\right)g\left(\tilde{\phi }\right)}}{2D^{\prime }\left(\tilde{\phi }\right)} \end{array}$$

and $\tilde{\phi }=-1/\beta .$ Both of them are outside of the region of interest. Moreover, given that the vertical axis of the *ϕv* plane is an invariant set of the system, the dynamics around them does not influence the dynamics on the region of interest.
